# Diffusion laws in dendritic spines

**DOI:** 10.1186/2190-8567-1-10

**Published:** 2011-10-27

**Authors:** David Holcman, Zeev Schuss

**Affiliations:** 1 Institute for Biology (IBENS), Group of Computational Biology and Applied Mathematics Ecole Normale Supérieure, 46 rue d'Ulm, 75005 Paris, France; 2 Department of Applied Mathematics, UMR 7598 Université Pierre et Marie Curie, Boite Courrier 187, 75252 Paris, France; 3 Department of Mathematics, Tel-Aviv University, Tel-Aviv 69978, Israel

## Abstract

Dendritic spines are small protrusions on a neuronal dendrite that are the main locus of excitatory synaptic connections. Although their geometry is variable over time and along the dendrite, they typically consist of a relatively large head connected to the dendritic shaft by a narrow cylindrical neck. The surface of the head is connected smoothly by a funnel or non-smoothly to the narrow neck, whose end absorbs the particles at the dendrite. We demonstrate here how the geometry of the neuronal spine can control diffusion and ultimately synaptic processes. We show that the mean residence time of a Brownian particle, such as an ion or molecule inside the spine, and of a receptor on its membrane, prior to absorption at the dendritic shaft depends strongly on the curvature of the connection of the spine head to the neck and on the neck's length. The analytical results solve the narrow escape problem for domains with long narrow necks.

## 1 Introduction

**Figure 1:**
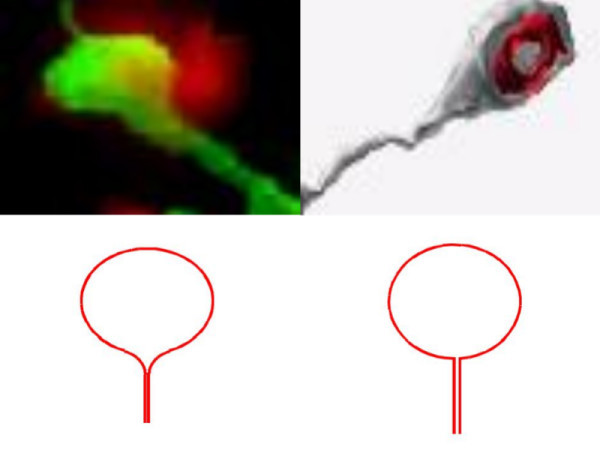
**Upper row: (from left to right) The dendritic spine head is connected smoothly to the neck (the postsynaptic density (PSD) is marked red) and (right) the connection is not smooth (from **[6]). **Lower row: **A mathematical idealization of a cross section: A cross section of a sharp and non-sharp connection approximating the spine morphology.

Recognized more than 100 hundreds years ago by Ramón y Cajal, dendritic spines are small terminal protrusions on neuronal dendrites, and are considered to be the main locus of excitatory synaptic connections. The general spine geometry consists of a relatively narrow cylindrical neck connected to a bulky head (the round part in Figure 1). Their geometrical shape correlates with their physiological function [1-6]. More than three decades ago, the spine-dendrite communication associated with the particle transfer was already anticipated [7] to be mediated not only by pure diffusion but it was hypothesized to involve other mechanisms such as twitching. This idea was reinforced by the findings [8] that inside the spine, the cytoplasmic actin is organized in filaments, involved in various forms of experimentally induced synaptic plasticity by changing the shape or volume of the pre- and postsynaptic side and by retracting and sprouting synapses. The fast dendritic spine contraction was finally confirmed in cultured hippocampal neurons [9] and consequences were studied theoretically in [10-12]. Interestingly, a serial electron microscopy and three-dimensional reconstructions of dendritic spines from Purkinje spiny branchlets of normal adult rats allowed to relate spine geometry to synaptic efficacy [1]. This image reconstruction approach leads to the conclusion that the cerebellar spine necks are unlikely to reduce transfer of synaptic charge by more than 5-20%, even if their smooth endoplasmic reticulum were to completely block passage of current through the portion of the neck that it occupies. The constricted spine neck diameter was proposed to isolate metabolic events in the vicinity of activated synapses by reducing diffusion to neighboring synapses, without significantly influencing the transfer of synaptic charge to the postsynaptic dendrite [1].

Change of spine morphology can be induced by synaptic potentiation protocols [13-15] and indeed intracellular signaling such as calcium released from stores alters the morphology of dendritic spines in cultured hippocampal neurons. These changes in geometry can affect the spine-dendrite communication. One of the first quantitative assessment of geometry was obtained by a direct measurement [16] of diffusion though the spine neck. Using photobleaching and photorelease of fluorescein dextran, by generating concentration gradients between spines and shafts in rat CA1 pyramidal neurons, the time course of re-equilibration was well approximated by a single exponential decay, with a time constant in the range of 20 to 100 ms. The role of the spine neck was further investigated using flash photolysis of caged calcium [3,17] and theory [18], and the main conclusion was that geometrical changes in the spine neck such as the length or the radius are key modulator of the spine-dendrite communication [12,19,20], affecting calcium dynamics. However, in all these studies, the nature of the connection between the neck and head was not considered. The theoretical studies [19,21] considered non-smooth connection only of the head to the narrow cylindrical neck (Figure 1) and did not account for any effect of curvature. This is precisely the goal of this article to investigate the consequences of this connection.

The connection between the head and the neck is not only relevant for the three-dimensional diffusion, but also essential to the analysis of other synaptic properties. Indeed, synaptic transmission and plasticity involve the trafficking of receptors [22-27] such as AMPA or NMDA receptors (AMPARs or NMDARs) that mediate the glutamatergic-induced synaptic current. Single particle approaches have further [28,29] revealed the heterogeneity of two-dimensional trajectories occurring on the neuron surface, suggesting that there are several biophysical processes involved in regulating the receptor motion. In addition, the number and type of receptors that shape the synaptic current [23] could be regulated by the spine geometry. This question was further explored theoretically [30,31], using asymptotic expressions for the residence time and experimentally [32] by monitoring the movements of AMPARs on the surface of mature neurons using FRAP. Employing a combination of confocal microscopy and photobleaching techniques in living hippocampal CA1 pyramidal neurons, a correlation between spine shape parameters and the diffusion and compartmentalization of membrane-associated proteins was recently confirmed [33]. Lateral diffusion seems to be a constitutive process of AMPAR trafficking; it depends on spine morphology and is restricted by the spine geometry [34].

**Figure 2:**
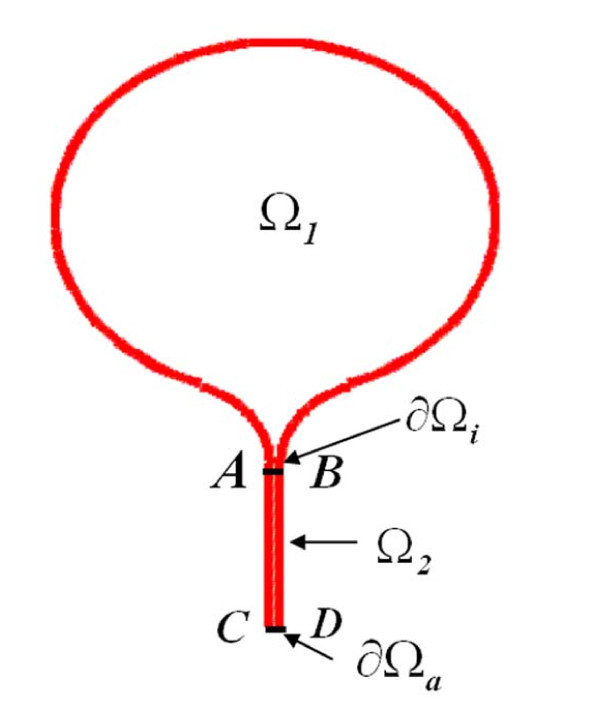
**The composite domain consists of a head Ω_1 _connected by an interface ∂Ω*_i _*to narrow neck Ω_2_**. The entire boundary is reflecting, except for a small absorbing part ∂Ω*_a_*.

In this article, we develop a method for computing the NET from composite spine-like structures that consist of a relatively large compartment (head) Ω_1 _and a narrow cylindrical neck of cross section |∂Ω*_a _*and length *L *(see Figure 2). Our connection formula is given as

(1) $${\bar{\tau }}_{x\to \partial \Omega_{a}}={\bar{\tau }}_{x\to \partial \Omega_{i}}+\frac{L^{2}}{2D}+\frac{|\Omega_{1}|L}{|\partial \Omega_{a}|D}.$$

The connection between the two parts in the context of the NET problem was attempted in [21,35] for the oversimplified model of a discontinuous connection. Here, we study a large class of connections and reveal the role of curvature in the spine-neck connection in regulating diffusion flux through narrow necks. More specifically, we study here the residence time of a Brownian particle from the spine head to the absorbing end of the spine neck moving either on the surface or inside the spine. We use the results of [36,37] for the mean first passage time (MFPT) to an absorbing boundary at the end of a cusp-shaped protrusion in the head. They account for the effects of curvature generated by the neck-head connection in the spine. The reciprocal of the MFPT is the rate of arrival (probability flux) of Brownian particles from the head to the dendrite [38]. We calculate the narrow escape time (NET) from spine-shaped domains with heads connected smoothly and non-smoothly to the neck.

## 2 The NET from a domain with a bottleneck

We consider two- and three-dimensional composite domains Ω that consist of a head Ω_1 _connected through a small interface ∂Ω*_i _*to a narrow cylindrical neck Ω_2_. The boundary of Ω is assumed reflecting to Brownian particles, except the far end of Ω_2_, denoted ∂Ω*_a_*, which is absorbing. For example, in Figure 2, the interface ∂Ω*_i _*is the black segment AB and the absorbing boundary ∂Ω*_a _*is the segment CD at the bottom of the strip. The NET from such a composite domain cannot be calculated by the methods of [39-42], because the contribution of the singular part of Neumann's function to the MFPT in a composite domain with a funnel or another bottleneck is not necessarily dominant. The method of matched asymptotic expansions requires different boundary or internal layers at a cusp-like absorbing window than at an absorbing window which is cut from a smooth reflecting boundary (see [43-46]). The methods used in [21,35] for constructing the MFPT in a composite domain of the type shown in Figure 1d are made precise here and the new method extends to the domains of the type shown in Figure 1c.

First, we recount some basic facts about the NET [35,39-41,43-45,47,48]. The NET is the MFPT of a Brownian trajectory to a small absorbing part of the boundary of a domain, whose remaining boundary reflects Brownian trajectories. Refined asymptotic formulas for the NET were derived in [42,46,49,50], and were used to estimated chemical reaction rates.

Consider Brownian motion ***x***(*t*) in a sufficiently regular bounded domain Ω, whose boundary ∂Ω consists of a reflecting part ∂Ω*_r _*and an absorbing part ∂Ω*_a_*. The expected lifetime of ***x***(*t*) in Ω, given ***x***(0) = ***x ***∈ Ω, is the MFPT *v*(***x***) of ***x***(*t*) from ***x ***to ∂Ω*_a _*is the solution of the mixed boundary value problem [38]

(2) $$\Delta v\left(x\right)=-\frac{1}{D}\;\text{for}\;x\in \Omega$$

(3) $$v\left(x\right)=0\;\text{for}\;x\in \partial \Omega_{a}$$

(4) $$\frac{\partial v\left(x\right)}{\partial n}=0\;\text{for}\;x\in \partial \Omega_{r},$$

where ∂*v*(***x***)/∂*n *is the normal derivative at the boundary point ***x***. If the size of the absorbing part ∂Ω*_a _*of the boundary is much smaller than the reflecting part ∂Ω*_r_*, the MFPT $\bar{\tau }=v\left(x\right)$ is to leading order independent of ***x ***∈ Ω*_a _*and can be represented by the Neumann function *N*(***x***, ***y***) as

(5) $$\bar{\tau }=v\left(y\right)=-\frac{1}{D}\int_{\Omega }N\left(x,y\right)dx-\int_{\partial \Omega_{a}}N\left(x,y\right)\frac{\partial v\left(x\right)}{\partial n}dS_{x}.$$

The sum of the integrals is independent of ***y ***∈ Ω*_a _*outside a boundary layer near ∂Ω*_a_*. The Neumann function is a solution of the boundary value problem

(6) $$\Delta_{x}N\left(x,y\right)=-\delta \left(x-y\right)+\frac{1}{|\Omega |}\;\text{for}\;x,y\in \Omega$$

(7) $$\frac{\partial N\left(x,y\right)}{\partial n_{x}}=0\;\text{for}\;x\in \partial \Omega ,y\in \Omega$$

and is defined up to an additive constant [39,47].

### 2.1 The MFPT from the head to the interface

In the two-dimensional case considered in [40] the interface ∂Ω*_i _*is an absorbing window cut from the smooth reflecting boundary of Ω_1_, as in Figure 1d. The MFPT ${\bar{\tau }}_{x\to \partial \Omega_{i}}$ is the NET from the reflecting domain Ω_1 _to the small interface ∂Ω*_i _*(of length *a*), such that *ε *= *π*|∂Ω*_i_*|/|∂Ω_1_| = *πa*/|∂Ω_1_| << 1 (this corrects the definition in [40]). It is given by

(8) $${\bar{\tau }}_{x\to \partial \Omega_{i}}=\frac{|\Omega_{1}|}{\pi D}\ln\frac{|\partial \Omega_{1}|}{\pi |\partial \Omega_{i}|}+O\left(1\right)\;\text{for}\;x\in \Omega_{1}\;\text{outside}\;\text{a}\;\text{boundary}\;\text{layer}\;\text{near}\;\partial \Omega_{i}.$$

In particular, if Ω_1 _is a disk of radius *R*, then for ***x ***= the center of the disk,

(9) $${\bar{\tau }}_{x\to \partial \Omega_{i}}=\frac{R^{2}}{D}\left[\log\frac{R}{a}+2\log2+\frac{1}{4}+O\left(\varepsilon \right)\right],$$

averaging with respect to a uniform distribution of ***x ***the disk

(10) $${\bar{\tau }}_{x\to \partial \Omega_{i}}=\frac{R^{2}}{D}\left[\log\frac{R}{a}+2\log2+\frac{1}{8}+O\left(\varepsilon \right)\right].$$

When the interface ∂Ω*_i _*(of length *a*) is located at an algebraic cusp with radius of curvature *R_c _*(see Figures 1c and 2), the MFPT is given in [36,37] as

(11) $$\bar{\tau }=\frac{|\Omega_{1}|}{4D\sqrt{2a/R_{c}}}\left(1+O\left(1\right)\right)\;\text{for}\;\varepsilon \ll |\partial \Omega |.$$

In the case of Brownian motion on a spherical head of the surface of revolution obtained by rotating the curve in Figure 1d about its axis, Ω_1 _is a sphere of radius *R *centered at the origin, connected to Ω_2 _by a circle ∂Ω*_i _*centered on the north-south axis near the south pole, with small radius *a *= *R *sin *δ*/2. The domain Ω_2 _is a right cylinder of radius *a *connected to Ω_1 _at ∂Ω*_i_*, and the absorbing boundary ∂Ω*_a _*is the circle of radius *a *at the bottom of the cylinder. The MFPT from Ω_1 _to ∂Ω*_i _*is given in [37,41,51-53] as

(12) $${\bar{\tau }}_{x\to \partial \Omega_{i}}=\frac{2R^{2}}{D}\log\frac{\sin\frac{\theta }{2}}{\sin\frac{\delta }{2}},$$

where *θ *is the angle between ***x ***and the south-north axis of the sphere.

**Figure 3:**
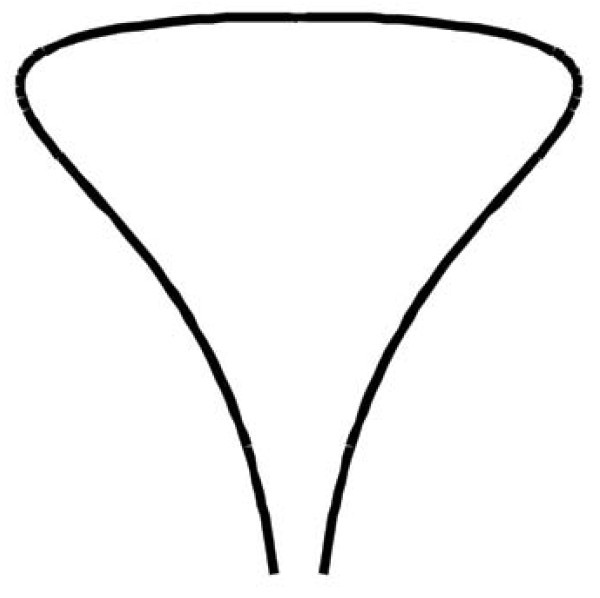
**A surface of revolution with a funnel**. The *z*-axis points down.

A surface of revolution generated by rotating a curve about an axis, as in Figure 3, with a funnel of diameter *ε *can be represented parametrically as

(13) x=r(z)cosθ,y=r(z)sin(θ),0≤θ<2π,0≤z<Λ,

where the axis of symmetry is the *z*-axis with *z *= 0 at the top of the surface and *z *= Λ at the end of the funnel, *r *is distance to the *z*-axis, and *r *= *r*(*z*) is the equation of the generating curve. We have

(14) $$\begin{array}{c} r\left(z\right)=O\left(\sqrt{z}\right)\;\text{near}\;z=0\\ r\left(z\right)=a+\ell^{-\alpha }{\left(\Lambda -z\right)}^{1+\alpha }\left(1+o\left(1\right)\right)\;\text{for}\;\alpha >0\;\text{near}\;z=\Lambda , \end{array}$$

where *ℓ *has dimension of length. For *α *= 1 the parameter *ℓ *is related to the radius of curvature *R_c _*at *z *= Λ by *ℓ *= 2*R_c_*. For *α *> 0 [37]

(15) $${\bar{\tau }}_{x\to \partial \Omega_{i}}\sim \frac{|\Omega_{1}|}{2D}\frac{{\left(\frac{\ell }{a}\right)}^{\alpha /1+\alpha }}{\left(1+\alpha \right)\sin\frac{\pi }{1+\alpha }},$$

where |Ω_1_| is the entire area of the surface. In particular, for *α *= 1 we get the MFPT

(16) $${\bar{\tau }}_{x\to \partial \Omega_{i}}\sim \frac{|\Omega_{1}|}{4D\sqrt{2a/R_{c}}}.$$

The case *α *= 0 corresponds to a circular cap of a small radius *a *cut from a closed surface.

The MFPT of Brownian motion from a solid ball Ω_1 _to a disk ∂Ω*_i _*of small radius *a *near the south pole is given by [42]

(17) $${\bar{\tau }}_{x\to \partial \Omega_{i}}=\frac{|\Omega_{1}|}{4aD}\left[1+\frac{a}{\pi R}\log\frac{R}{a}+o\left(\frac{a}{R}\log\frac{R}{a}\right)\right]$$

(note that the MFPT is independent of ***x ***up to second order, see [46]). For a general three-dimensional domain, Ω_1 _the MFPT to a circular cap ∂Ω*_i _*cut from a smooth boundary is given by [42]

(18) τ¯x→∂Ωi=∣Ω1∣4aD1+Lx+Rx2π∂Ωiπ1∕2 log∂Ω1∂Ωi+o∂Ωi∂Ω1 log∂Ωi∂Ω1+O(1)D,

where *L**_x_***, *R**_x _***are the principal curvatures at a point ***x***, and |∂Ω*_i_*| = *πa*^2 ^is the area of the circular cap.

When the interface ∂Ω*_i _*is a circular disk of radius *a *at the end of an axisymmetric solid funnel, the MFPT is drastically affected and changes to

(19) $${\bar{\tau }}_{x\to \partial \Omega_{i}}=\frac{1}{\sqrt{2}}{\left(\frac{R_{c}}{a}\right)}^{3∕2}\frac{|\Omega_{1}|}{R_{c}D}\left(1+o\left(1\right)\right)\;\text{for}\;a\ll R_{c},$$

where *R_c _*is the radius of curvature at the end of the funnel [37].

## 3 Connecting a head to a narrow neck

We consider Brownian motion in a domain Ω that consists of a head, which is a regular bounded domain Ω_1_, and a narrow neck Ω_2_, which is a right circular or planar cylinder of length *L*, perpendicular to the boundary ∂Ω_1_, and of radius *a *(see Figure 2). Thus, the interface ∂Ω*_i _*between the head and the neck is a line segment, a circle, or a circular disk, depending on the dimension. We assume that ∂Ω_1 _- ∂Ω*_i _*is reflecting and that the other basis of the neck, ∂Ω*_a _*⊂ ∂Ω_2_, is absorbing for the Brownian motion. The length (or area) |∂Ω*_i_*| is given by

(20) $$|\partial \Omega_{i}|=\left\{\begin{array}{cc} a & \text{for}\;\text{a}\;\text{line}\;\text{segment}\\ 2\pi a & \text{for}\;\text{a}\;\text{circle}\\ \pi a^{2} & \text{for}\;\text{a}\;\text{disk}. \end{array}\right)$$

The MFPT ${\bar{\tau }}_{x\to \partial \Omega_{a}}$ can be represented as [54], [[38], Lemma 10.3.1, p. 388]

(21) $${\bar{\tau }}_{x\to \partial \Omega_{a}}={\bar{\tau }}_{x\to \partial \Omega_{i}}+{\bar{\tau }}_{\partial \Omega_{i}\to \partial \Omega_{a}},$$

where the MFPT ${\bar{\tau }}_{\partial \Omega_{i}\to \partial \Omega_{a}}$ is ${\bar{\tau }}_{x\to \partial \Omega a}$, averaged over ∂Ω*_i _*with respect to the flux density of Brownian trajectories in Ω_1 _into an absorbing boundary at ∂Ω*_i _*(see [38] for further details).

First, we calculate ${\bar{\tau }}_{\partial \Omega_{i}\to \partial \Omega_{a}}$ and the absorption flux at the interface. In the narrow neck Ω_2 _the boundary value problem (2)-(4) can be approximated by the one-dimensional boundary value problem

$$\begin{array}{c} Du_{zz}=-1\\ u\left(0\right)=0,\;u\left(L\right)=u_{H}, \end{array}$$

where the value at the interface *u*(*L*) = *u_H _*is yet unknown. The solution is given by

(22) $$u\left(z\right)=-\frac{z^{2}}{2D}+Bz,$$

so that

(23) $$u\left(L\right)=u_{H}=-\frac{L^{2}}{2D}+BL,$$

which relates the unknown constants *B *and *u_H_*. The constant *B *is found by multiplying Equation 2 by the Neumann function *N*(***x***, ***y***), integrating over Ω_1_, applying Green's formula, and using the boundary conditions (3) and (4). Specifically, we obtain for all ***y ***∈ ∂Ω*_i_*

(24) $$v\left(y\right)=-\frac{1}{D}\int_{\Omega_{1}}N\left(x,y\right)dx-\int_{\partial \Omega_{i}}N\left(x,y\right)\frac{\partial v\left(x\right)}{\partial n}dS_{x}+\frac{1}{|\Omega_{1}|}\int_{\Omega_{1}}v\left(x\right)dx.$$

Approximating, as we may, *v*(***y***) ≈ *u*(*L*) and using (23), we obtain

(25) $$-\frac{L^{2}}{2D}+BL=-\frac{1}{D}\int_{\Omega_{1}}N\left(x,y\right)dx-\int_{\partial \Omega_{i}}N\left(x,y\right)\frac{\partial v\left(x\right)}{\partial n}dS_{x}+\frac{1}{|\Omega_{1}|}\int_{\Omega_{1}}v\left(x\right)dx.$$

Because *v*(***x***) is the solution of the boundary value problem (2)-(4) in the entire domain Ω = Ω_1 _⋃ Ω_2_, the meaning of (25) is the connecting rule (21), where

(26) $${\bar{\tau }}_{x\to \partial \Omega_{a}}=\frac{1}{|\Omega_{1}|}\int_{\Omega_{1}}v\left(x\right)dx$$

(27) $${\bar{\tau }}_{\partial \Omega_{i}\to \partial \Omega_{a}}=u\left(L\right)$$

(28) $${\bar{\tau }}_{x\to \partial \Omega_{i}}=-\frac{1}{D}\int_{\Omega }N\left(x,y\right)dx-\int_{\partial \Omega_{i}}N\left(x,y\right)\frac{\partial v\left(x\right)}{\partial n}dS_{x}.$$

Equation 26 gives the MFPT, averaged over Ω_1_. The averaging is a valid approximation, because the MFPT to ∂Ω*_i _*is constant to begin with (except in a negligible boundary layer). Equation 27 is the MFPT from the interface to the absorbing end ∂Ω*_a _*of the strip, and (28) follows from (5).

Matching the solutions in Ω_1 _and Ω_2 _continuously across ∂Ω*_i_*, we obtain the total flux on ∂Ω*_i _*as

(29) $$J=D\int_{\partial \Omega_{i}}\frac{\partial v\left(x\right)}{\partial \nu }dS_{x}=-\left(|\Omega_{1}|\;+\;|\Omega_{2}|\right),$$

Noting that ∂*v*(***x***)/∂*n *= -*u*'(0) = -*B*, we get from (20) and (29) that

(30) $$B=-\left\{\begin{array}{cc} \frac{|\Omega_{1}|}{aD}+\frac{L}{D} & \text{for}\;\text{a}\;\text{line}\;\text{segment}\\ \frac{|\Omega_{1}|}{2\pi aD}+\frac{L}{D} & \text{for}\;\text{a}\;\text{circle}\\ \frac{|\Omega_{1}|}{\pi a^{2}D}+\frac{L}{D} & \text{for}\;\text{a}\;\text{circular}\;\text{disk}. \end{array}\right)$$

Finally, we put (21)-(30) together to obtain

(31) $${\bar{\tau }}_{x\to \partial \Omega_{a}}={\bar{\tau }}_{x\to \partial \Omega_{i}}+\frac{L^{2}}{2D}+\frac{|\Omega_{1}|L}{|\partial \Omega_{a}|D}.$$

The MFPT ${\bar{\tau }}_{x\to \partial \Omega_{i}}$ is given by (8)-(19) above.

### 3.1 The NET from two- and three-dimensional domains with bottlenecks

The expression (31) for the NET from a domain with a bottleneck in the form of a one-dimensional neck, such as a dendritic spine, can be summarized as follows. Consider a domain Ω with head Ω_1 _and a narrow cylindrical neck Ω_2 _of length *L *and radius *a*. The radius of curvature at the bottleneck in smooth connecting funnel is *R_c_*. In the two-dimensional case

(32) $${\bar{\tau }}_{x\to \partial \Omega_{a}}=\left\{\begin{array}{c} \frac{|\Omega_{1}|}{\pi D}\ln\frac{|\partial \Omega_{1}|}{a}+\frac{O\left(1\right)}{D}+\frac{L^{2}}{2D}+\frac{|\Omega_{1}|L}{aD}\\ \text{planar}\;\text{spine}\;\text{connected}\;\text{to}\;\text{the}\;\text{neck}\;\text{at}\;\text{a}\;\text{right}\;\text{angle}\\ \frac{\pi |\Omega_{1}|}{D}\sqrt{\frac{R_{c}}{a}}\left(1+o\left(1\right)\right)+\frac{L^{2}}{2D}+\frac{|\Omega_{1}|L}{2\pi aD}\\ \text{planar}\;\text{spine}\;\text{with}\;\text{a}\;\text{smooth}\;\text{connecting}\;\text{funnel}\\ \frac{|\Omega_{1}|}{2\pi D}\log\frac{\sin\frac{\theta }{2}}{\sin\frac{\delta }{2}}+\frac{L^{2}}{2D}+\frac{|\Omega_{1}|L}{2\pi aD}\\ \text{spherical}\;\text{spine}\;\text{connected}\;\text{to}\;\text{the}\;\text{neck}\;\text{at}\;\text{a}\;\text{right}\;\text{angle}\\ \frac{|\Omega_{1}|}{2D}\frac{{\left(\frac{\varepsilon }{\ell }\right)}^{-\alpha ∕1+\alpha }2^{\alpha ∕1+\alpha }}{\left(1+\alpha \right)\sin\frac{\pi }{1+\alpha }}+\frac{L^{2}}{2D}+\frac{|\Omega_{1}|L}{2\pi aD}\\ \text{spherical}\;\text{spine}\;\text{with}\;\text{a}\;\text{smooth}\;\text{connecting}\;\text{funnel,} \end{array}\right)$$

where *R *is the radius of the sphere, *a *= *R *sin *δ*/2, and *θ *is the initial elevation angle on the sphere. If |Ω_1_| >>*aL *and *L *>>*a*, the last term in (32) is dominant, which is the manifestation of the many returns of Brownian motion from the neck to the head prior to absorption at ∂Ω*_a _*(see an estimate in [19]). The last line of (32) agrees with the explicit calculation in [37].

The NET of a Brownian motion from a three-dimensional domain Ω with a bottleneck in the form of a narrow circular cylinder of cross-sectional area *πa*^2 ^is given by

**Figure 4:**
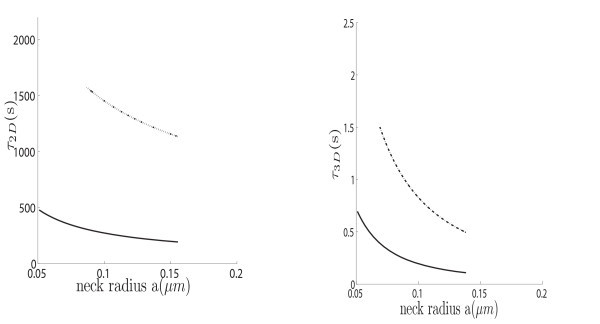
**Left: The NET of Brownian motion on a sphere with a bottleneck connected by a smooth funnel to the neck (dashed line), and with a non-smooth connection (continuous line)**. **Right: **The NET of Brownian motion in a ball with a bottleneck connected by a smooth funnel to the neck (dashed line), and with a non-smooth connection (continuous line).

**Figure 5:**
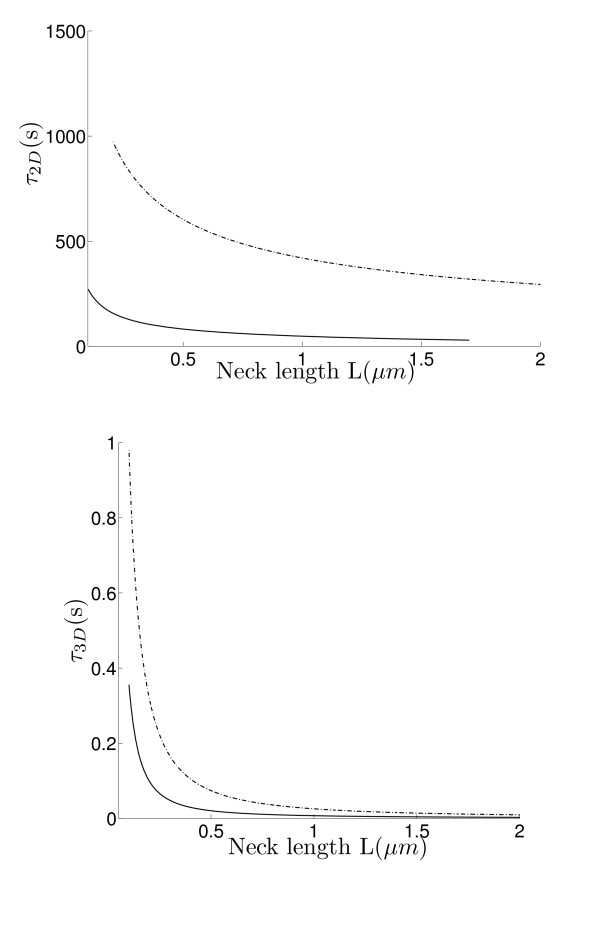
**Effect of modulating the neck length**. The geometry is the same as in Figure 5.

(33) τ¯x→∂Ωa=∣Ω1∣4aD1+aπRlogRa+O(1)D+L22D+∣Ω1∣Lπa2DsolidsphericalheadofradiusRconnectedtotheneckatarightangle∣Ω1∣4aD1+(Lx+Rx)2π∂Ωaπ1∕2 log|∂Ω1||∂Ωa|+o∂Ωa∂Ω1 log∂Ωa∂Ω1+O(1)D+L22D+∣Ω1∣Lπa2Dageneralheadconnectedtotheneckatarightangle12Rca3∕2∣Ω1∣RcD(1+o(1))+L22D+∣Ω1∣Lπa2Dageneralheadconnectedsmoothlytotheneckbyafunnel,

where *R_c _*is the curvature at the cusp. The order 1 term can be computed for the sphere using the explicit expression of the Neumann-Green function [46]. Figures 4 and 5 show the NET for various parameters, such as the neck length and radius.

Finally, the influence of the neck length on the residence time is shown in Figure 5: changing the neck length modulates dramatically the residence time. Interestingly, the geometry of the connection affects much significantly the dimension two rather than that the three dimensional Brownian motion.

## Discussion and conclusion

We have shown that the mean residence time (or flux) of Brownian particles inside a spine-like structure or on its surface depends strongly on the geometrical properties of both head and neck. Surprisingly, it also depends strongly on the smoothness of the connection between the two.

The application to a freely diffusing AMPA receptor, which is responsible for the excitatory synaptic current, shows that its motion on the spine membrane is strongly restricted by dendritic spine geometry. Our results can be used to estimate the residence time of the receptor on the membrane if interactions with any scaffolding molecules are neglected (the latter are mostly concentrated in a local microdomain called the PSD). Using Equation 32 for non-smooth geometry (Figure 1b) and for a spherical head of radius *R *= 1 *μ*m, a neck length *L *= 1 *μ*m, a neck radius *a *= 0.1 *μ*m, and a diffusion coefficient *D *= 0.1 *μ*m^2^/*s*, we obtain from the third line of Equation 32 that the residence time is *τ*_2*D *_≈ 260 s, while for a smooth connecting geometry (1a) with a curvature radius of *R_c _*= 1 *μ*m at the connecting neck-head *τ*_2*D *_≈ 1150 s (line 2 in Equation 32). Evidently, the residence time is more than doubled, leading to the conclusion that there is a significant difference between the function of spines with smoothly and non-smoothly connected necks. We conclude from this analysis that an AMPA receptors that do not interact with the PSD stay on a typical dendritic spine between one and a half to 5 min on average and this residence time is controlled mostly by the geometrical properties of the spine.

We now consider the residence time for freely diffusing particles such as molecules, mRNA, and ions (e.g., calcium) inside a dendritic spine. For a calcium ion, the diffusion constant is about *D*_ca _= 400 *μ*m^2^/s [12]. Calcium ions that exit the spine only to the dendrite shaft at the end of the neck, but not through exchangers, give the following residence time estimates. Using formula 33 for a non-smooth connection between the spine head and the neck, we obtain that *τ*_3*D *_≈ 195 ms (line 1), while for a smooth connection with radius of curvature 1 *μ*m, the residence time is *τ*_3*D *_≈ 820 ms (line 3 in Equation 33). Interestingly, the mean residence time is tripled from the non-smooth to a smooth connection.

A remaining open question is to extend the present analysis to the case where many binding molecules can trap receptors. This effect should be expected to significantly increase the residence time inside a dendritic spine, as has already been observed in [55] for the case of a receptor inside the PSD. The present mathematical analysis of the residence time provides a solution to the narrow escape problem for domains with bottlenecks [21,35]. Other generalizations of this study is to include the dynamics of many receptors [30,56] or/and to study dendritic trafficking [57].

There are many other factors that affect the spine-dendrite communication with respect to calcium. This includes calcium pumps, endogenous buffers, calcium stores, the number and rates of exchangers. These mechanisms affect the residence of calcium in spines [58-60] and it would be interesting to add them in the present analysis.

## Competing interests

The authors declare that they have no competing interests.

## Authors' contributions

DH and ZS contributed equally to the manuscript. All authors read and approved the final manuscript.
